# Randomized Cross-Sectional Study to Compare HIV-1 Specific Antibody and Cytokine Concentrations in Female Genital Secretions Obtained by Menstrual Cup and Cervicovaginal Lavage

**DOI:** 10.1371/journal.pone.0131906

**Published:** 2015-07-06

**Authors:** Derseree Archary, Lenine J. Liebenberg, Lise Werner, Sahil Tulsi, Nelisile Majola, Nivashnee Naicker, Sarah Dlamini, Thomas J. Hope, Natasha Samsunder, Salim S. Abdool Karim, Lynn Morris, Jo-Ann S. Passmore, Nigel J. Garrett

**Affiliations:** 1 Centre for the AIDS Programme of Research in South Africa, Nelson R. Mandela School of Medicine, University of KwaZulu–Natal, Durban, South Africa; 2 Department of Cell & Molecular Biology, Northwestern University Feinberg School of Medicine, Chicago, Illinois, United States of America; 3 Department of Epidemiology, Mailman School of Public Health, Columbia University, New York, NY, United States of America; 4 National Institute for Communicable Diseases, Johannesburg, South Africa; 5 Infectious Disease and Molecular Medicine, University of Cape Town, Observatory, Cape Town, South Africa; 6 National Health Laboratory Services, Cape Town, South Africa; Burnet Institute, AUSTRALIA

## Abstract

**Introduction:**

Optimizing methods for genital specimen collection to accurately characterize mucosal immune responses is a priority for the HIV prevention field. The menstrual cup (MC) has been proposed as an alternative to other methods including cervicovaginal lavage (CVL), but no study has yet formally compared these two methods.

**Methods:**

Forty HIV-infected, antiretroviral therapy-naïve women from the CAPRISA 002 acute HIV infection cohort study were randomized to have genital fluid collected using the MC with subsequent CVL, or by CVL alone. Qualitative data, which assessed levels of comfort and acceptability of MC using a 5-point Likert scale, was collected. Luminex multiplex assays were used to measure HIV-specific IgG against multiple gene products and 48 cytokines.

**Results:**

The majority (94%) of participants indicated that insertion, wearing and removal of the MC was comfortable. Nineteen MCs with 18 matching, subsequent CVLs and 20 randomized CVLs were available for analysis. Mucosal IgG responses against four HIV-antigens were detected in 99% of MCs compared to only 80% of randomized CVLs (p = 0.029). Higher specific antibody activity and total antibodies were observed in MCs compared to CVL (all p<0.001). In MCs, 42/48 (88%) cytokines were in the detectable range in all participants compared to 27/48 (54%) in CVL (p<0.001). Concentrations of 22/41 cytokines (53.7%) were significantly higher in fluid collected by MC. Both total IgG (r = 0.63; p = 0.005) and cytokine concentrations (r = 0.90; p<0.001) correlated strongly between MC and corresponding post-MC CVL.

**Conclusions:**

MC sampling improves the detection of mucosal cytokines and antibodies, particularly those present at low concentrations. MC may therefore represent an ideal tool to assess immunological parameters in genital secretions, without interfering with concurrent collection of conventional CVL samples.

## Introduction

Elucidation of local immune responses in the genital mucosa is key to informing the design of effective biomedical interventions that prevent the spread of HIV and other sexually transmitted infections (STIs). Given the heterogeneity in the quality of mucosal samples obtained through different sampling procedures, methods improving the detection of immune mediators and soluble immune markers in the cervicovaginal compartment need to be optimized and verified across different studies and settings [1]. The benefits and shortfalls of several methods for mucosal sample collection have been compared [2–7] and newer methods of genital mucosal sampling require rigorous comparison with conventional methods for the quantification of antibodies, soluble proteins, and innate anti-microbial factors.

Swab, sponge or cervicovaginal lavage (CVL) sampling is among the conventional methods used to collect cervicovaginal secretions. Specimens collected by Weck-cells, sno-strips, or ophthalmic sponges yield consistently higher concentrations of immune markers than CVL sampling [3]. Generally, sponges have been shown to yield higher concentrations of measured markers compared to more dilute specimen types such as CVL, but are also subject to a high degree of variability in the amount of genital fluid collected [2,8,9]. Even among the various types of sponges used to sample the cervix, certain sponges were shown to have superior sample recovery [10,11].

Several factors that should be considered for optimal sample collection include reproducibility, biological representativeness, minimum sample dilution, level of discomfort, invasiveness to the participant, ease of collection, and optimal recovery of target proteins, or cells from the collection apparatus [8]. Genital mucosal self-sampling using menstrual cups (MC) or other novel devices has been reported [7,12–15] and may be an attractive additional method or replacement to CVL as it simplifies the collection procedure, and may circumvent the need for clinician-driven genital sampling. Additionally, MC was well accepted and tolerated by clinical trial participants who consented to a protocol requiring repeated mucosal sampling [6].

This randomized study compared the utility of MC and CVL sampling by assessing qualitative data on comfort and acceptability of MC versus CVL, quantifying in each the immune factors associated with HIV, namely HIV-specific antibody titres and cytokine concentrations, and the influence of mucosal sampling order on the detection of these markers.

## Methods

### Study population and design

Since 2004, the CAPRISA 002 study has been following women from acute HIV infection at two CAPRISA Clinical Research Sites in KwaZulu-Natal, South Africa [16]. On routine CAPRISA 002 visits, 40 HIV-infected, antiretroviral therapy naïve women were offered to participate in this randomized controlled study. Participants provided additional written informed consent for the study. Women who were pregnant, menstruating, using intra-uterine devices, or showed symptoms or signs of genitourinary tract infections were excluded from the study.

**Fig 1:**
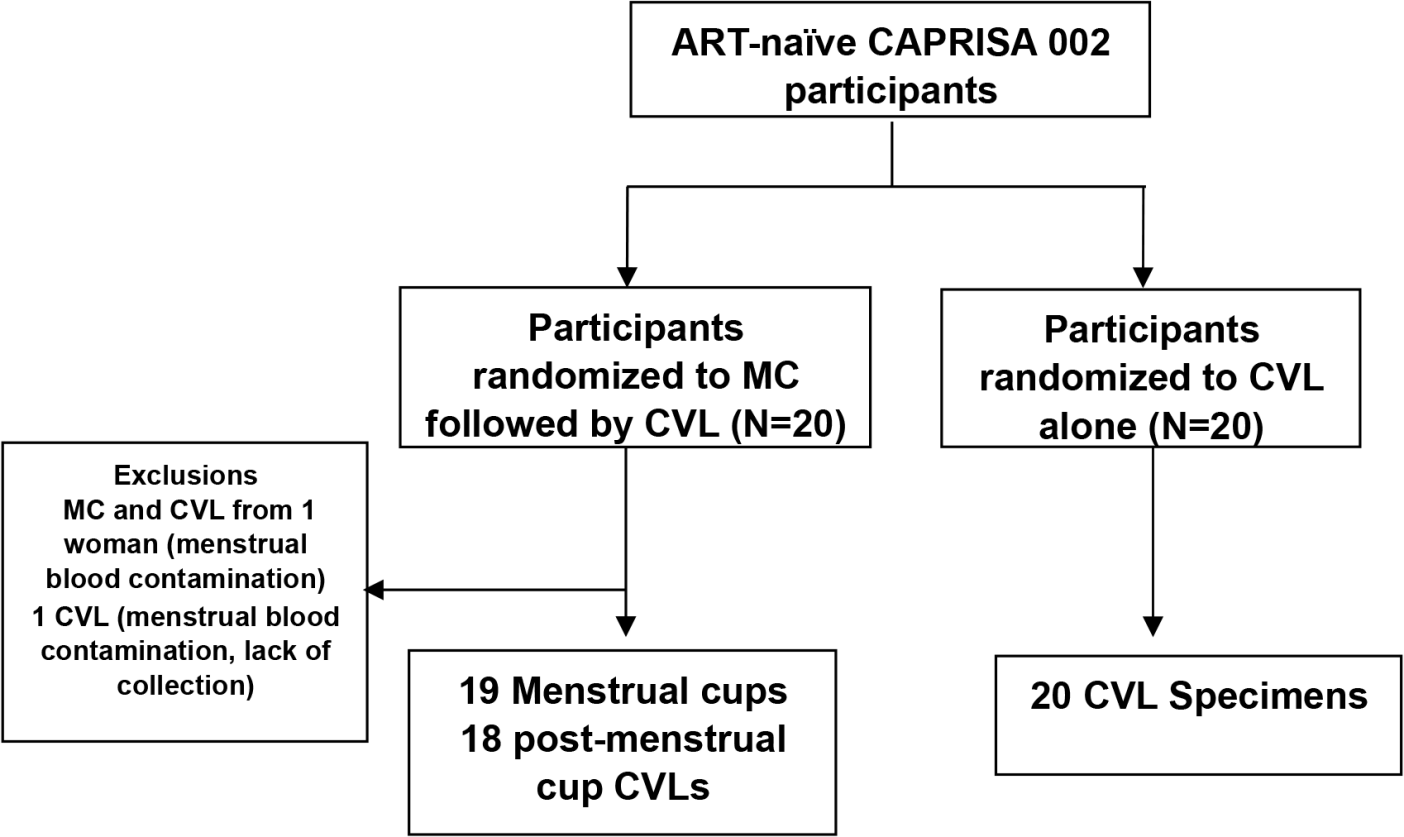
Schema of study design.

Twenty women were randomized to the MC (SoftCup, EuroFemPro, Netherlands, or the SoftCup, Instead Inc., San Diego, CA) arm (Fig 1). This involved the vaginal insertion of a MC by either a study clinician or nurse for a period of two hours. During this two hour period with the inserted MC, participants proceeded with the routine study specific procedures including for e.g. blood draws and clinical examination. Immediately after assisted removal of the MC by the health professional, routine collection of CVL was performed. Participants carried on with their routine study visit during this time. In the MC arm, one participant was excluded from further evaluation due to menstrual blood contamination, and another participant did not provide a CVL specimen. Therefore, there were a total of 19 MC arm samples and 18 with matching post-MC CVLs. A further 20 women were randomized to the CVL only arm of the study, the current standard of sample collection in the CAPRISA 002 study. Questionnaires with 5-point Likert scales describing levels of comfort and acceptability were distributed to all women from the MC arm after the procedure, and were collected after completion by the participants.

### Ethics Statement

The CAPRISA 002 study and this sub-study were both approved by the University of KwaZulu-Natal Biomedical Research Ethics Committee (Ref: E013/04 and BE114/12).

### Menstrual cup and CVL collection and processing

After MCs were removed, they were placed into a labelled sterile 50ml conical tube and were transported on ice to the CAPRISA Mucosal Immunology Laboratory for further processing within 4–6 hours of collection. The MC was centrifuged at 2,000 rpm for 10 minutes at room temperature [17]. The MC secretions were centrifuged as before to separate the mucous pellet from the fluid supernatant. If the fluid supernatant dispersed into the solid mucous pellet, the samples were centrifuged a third time. Phosphate buffered saline (PBS), was used to dilute the clear supernatant fluid of the MC five-fold. A volume of 50 μl of the fluid supernatant obtained after centrifugation was diluted with 200μl of PBS before storage at -80°C. On thawing, a further five-fold dilution of sample was conducted using PBS, and the resultant 25-fold dilution from the MC supernatant fraction was used in antibody and cytokine multiplex assays.

The CVLs were obtained and processed as previously reported [18,19]. Briefly, 5 ml of sterile normal saline was used to bathe the cervix. The pooled fluids were subsequently aspirated using a sterile plastic bulb pipette and dispensed into sterile conical tubes. Specimens were transported on ice to the laboratory within 4–6 hours of collection. CVLs were centrifuged, and the supernatants of approximately 1 ml each were stored at −80°C.

### HIV-1-specific binding antibody assay

Antibodies against four HIV proteins were measured using a customized HIV-1 binding antibody multiplex assay (BAMA) [20], which included: consensus subtype C gp120, gp41 (ImmunoDX, USA), p66 (RT) and p24 Gag (Protein Sciences Corporation, USA). A total of 5×10^6^ carboxylated fluorescent beads (Bio-Rad Laboratories, Inc; Hercules, Ca, USA) were covalently coupled to 25 μg of one of the purified HIV antigens and incubated with 1:50, 1:500, 1:1000 and 1:2000 dilutions of the MC specimens, or 1:20 up to 1:200 dilutions of the CVLs for detection of HIV-1 specific IgGs. These dilutions were necessary in order to detect the HIV-specificities in the linear range of the standard curve. HIV-specific antibody IgGs were detected with goat anti-human IgG (Southern Biotech, Birmingham, AL) conjugated to phycoerythrin at 4 μg/ml. Beads were then washed and antibody measurements were acquired on a Bio-Plex 200 multiplex system (Bio-Rad Laboratories, Inc; Hercules, Ca, USA), and was reported as a mean fluorescent intensity (MFI). Background values (beads in the absence of detection antibody) and normal human plasma were utilized as negative controls. All assays were run under good clinical laboratory practice-compliant conditions, including tracking of positive controls by Levy-Jennings charts consistent with other studies using customized BAMA [21–23]. Positivity criteria for antibody-antigen pairs were pre-determined using a set of CVLs from 30 HIV-1 negative individuals [MFI standard deviation of 3] and a cut-off value of 100 MFI thereafter determined a positive result.

### IgG isotyping, total immunoglobulin quantification and HIV specific activity

To control for the inter-subject variation in genital fluid recovery and normalize to total immunoglobulin concentrations, total IgG concentrations were measured in CVL samples. Although MC collection volumes were less variable, total IgG levels were also measured in these samples. Antibody subclasses IgG1, IgG2, IgG3, and IgG4 were quantified using total antibody isotyping kits (Bio-Rad Laboratories, Inc; Hercules, Ca, USA) according to the manufacturer’s instructions, and the levels were determined by MFI plate readout on the Bio-Plex 200 multiplex system). MC-derived supernatants were diluted from 1:200 up to 1:2000, and up to 1:200 for CVLs in PBS to ensure that the MFIs for the HIV-specific antibodies were detected in the linear range of the standard curve. HIV-1 specific IgG activity in the CVL was calculated by determining the total amount of immunoglobulin in the assay for IgG1, IgG2, IgG3 and IgG4 in ng/ml for each genital sample. Specific activity, the ratio of the MFI for each HIV-specific IgG over total Ig (ng/ml), was calculated and adjusted for the dilution factor [log_10_ (MFI/ng ml^-1^)]. The MFI obtained for the HIV-1 specific IgG was then divided by the total immunoglobulin amount (MFI*dilution/ng/ml) to give the IgG specific activity in order to adjust for varying recovery volumes when performing CVLs. In addition, a second level cut-off for specific activity, based on HIV seronegative individuals, was applied to assess a positive response in the genital tract. Values falling below a detectable specific activity cut-off were included in the analysis and assigned a value as one tenth of the value of the specific activity cut-off for each HIV-specific IgG.

### Measurement of genital cytokine concentrations

The concentrations of 48 cytokines (Bio-Plex Pro Human Cytokine Group I 27-Plex Panel and Group II 21-Plex Panel; Bio-Rad Laboratories, Inc; Hercules, Ca, USA) were measured in MC and CVL specimens by Luminex multiplexing technology. Cytokines were measured in undiluted CVLs, and in a final 25-fold dilution in MC samples. The cytokine panel included the pro-inflammatory, haematopoietic, regulatory, adaptive, and/or growth-related cytokines: interleukin (IL)-1β, IL-1Rα, IL-2, IL-4, IL-5, IL-6, IL-7, IL-8, IL-9, IL-10, IL-12p70, IL-12p40, IL-16, IL-18, IL-1α, IL-2Rα, IL-3, IL-13, IL-15, IL-17, basic fibroblast growth factor (FGF), cutaneous T-cell attracting chemokine (CTACK), eotaxin, granulocyte colony-stimulating factor (G-CSF), granulocyte macrophage colony-stimulating factor (GM–CSF), growth regulated (GRO)-α, hepatocyte growth factor (HGF), interferon (IFN)-γ, IFN-α2, interferon gamma-induced protein (IP)-10, leukemia inhibitory factor (LIF), monocyte chemotactic protein (MCP)-1, MCP-3, macrophage colony-stimulating factor (M-CSF), monokine induced by gamma-Interferon (MIG), macrophage migration inhibitory factor (MIF), macrophage inflammatory protein (MIP)–1α, MIP-1β, nerve growth factor (NGF)-β, platelet derived growth factor (PDGF)-ββ, regulated upon activation normal T cell expressed and presumably secreted (RANTES), stem cell factor (SCF), stem Cell Growth Factor (SCGF)-β, stromal derived factor (SDF)-1α, tumor necrosis factor (TNF)–α, TNF-β, TNF-related apoptosis inducing ligand (TRAIL), and vascular endothelial growth factor (VEGF) measured using Bio-Plex Pro Human Cytokine kits and a Bio-Plex MagPix Array Reader (Bio-Rad Laboratories). The sensitivity of these kits ranged between 0.2 and 45.2 pg/ml for each of the 48 cytokines measured. Data was collected using Bio-Plex Manager software version 6, and a 5 PL regression formula was used to calculate sample concentrations from the standard curves. Cytokine levels below the lower limit of detection (LLOD) of the assay were reported as the mid-point between the lowest concentration measured for each cytokine and zero. To minimize the effect of inter-plate variability, MC and CVL specimens from the same participant were analyzed on the same plate.

### Statistical analysis

Basic descriptive statistics were used to characterize the participants. Differences between arms were compared using Fisher’s exact test for categorical data or Mann-Whitney tests for continuous data. Comparisons of HIV antibody specificities between groups were performed using either Mann-Whitney tests for independent comparisons or Wilcoxon signed rank test for paired samples. Fisher’s exact test was used to compare detectability of cytokine responses between MC and randomized CVL samples. Pearson’s correlation was used to determine associations between the matched MC and CVL samples, where values were log transformed to ensure normality. The false discovery rate method was used to correct for multiple comparisons for both antibody and cytokine comparisons. P-values less than 0.05 were considered significant. Statistical analysis was performed using SAS version 9.3 (SAS Institute Inc., Cary) and graphs were drawn using GraphPad Prism 6 software.

## Results

### Baseline characteristics

The median age for women in the study was 29 years (interquartile range (IQR) 26–32 years) and women had been HIV-infected for a median 60 months (IQR 51–70 months). Median CD4 count and viral load at randomization were 558 cells/μl (IQR 448–734 cells/μl) and 4.05 log copies/ml (IQR 3.51–4.42 log copies/ml). Participant characteristics by study arm are summarized in Table 1. There were no significant differences in age, time post-infection, use of injectable contraception, CD4+ T cell counts, viral loads, or sexually transmitted infections between women randomized to either receive a CVL alone or MC followed by a CVL.

**Table 1:** Characteristics of participants overall, and stratified by study arm.

Characteristic	Overall (N = 39)	MC arm (N = 19)	CVL only arm (N = 20)	p-value
Age (years); median (IQR)	29 (26–32)	29 (26–32)	28 (26–32)	0.502
Months post Infection; median (IQR)	60 (51–70)	60 (48–70)	60 (54–70)	0.559
CD4 T cell count (cells/μ); median (IQR)	558 (448–734)	606 (494–765)	507 (439–701)	0.367
Viral load (log copies/ml); median (IQR)	4.05 (3.51 4.42)	3.71 (2.55–4.47)	4.08 (3.72–4.38)	0.312
Injectable hormonal contraception; % (n)	56.4% (22)	47.4% (9)	65.0% (13)	0.341
Bacterial vaginosis; % (n/N)	14.3% (3/21)	27.3% (3/11)	0.0% (0/10)	0.214
*T*. *vaginalis*; % (n/N)	4.8% (1/21)	9.1% (1/11)	0.0% (0/10)	1.000
*N*. *gonorrhoeae*; % (n/N)	4.8% (1/21)	9.1% (1/11)	0.0% (0/10)	1.000
*C*. *trachomatis*; % (n/N)	19.1% (4/21)	27.3% (3/11)	10.0% (1/10)	0.587
*M*. *genitalium*; % (n/N)	14.3% (3/21)	9.1% (1/11)	20.0% (2/10)	0.589

Not all the study participants were tested for an STI or BV

Of the 19 women in the MC arm, nine (47.4%) were on depot medroxyprogesterone acetate (DMPA) injectable contraception, one participant had a tubal ligation, while the remaining nine women (47.4%) were not on any hormonal contraception. Of the 20 women in the CVL only arm, 13 (65.0%) were on injectable contraception [DMPA (n = 11) and norethisterone (n = 2)], while seven (35.0%) reported no contraceptive use (including one woman in menopause). Of the 22 women on injectable contraception, irrespective of study arm, 15 (68.2%) reported amenorrhoea.

### Feasibility and Acceptability of Menstrual Cup Sampling

The median time that the MC was in place was 120 minutes (range 88–175 minutes). A total of 17/20 (85%) women in the MC arm returned the questionnaires about acceptability of the MC sampling method. All of the women opted for the nurse or clinical practitioner to insert and remove the MC. A majority (94%, 16/17) of participants answered that clinician insertion was acceptable, that wearing the MC and removal was comfortable. All participants indicated their willingness to wear a MC again, and all preferred to have the MC inserted instead of a speculum. Nearly one third (29%) indicated that they would consider self-inserting the MC if adequately trained, with 47% being unsure and 24% indicating that they did not want to self-insert the MC.

### Menstrual cup specimens yield higher HIV-1 specific and total antibody levels than CVL specimens

**Fig 2:**
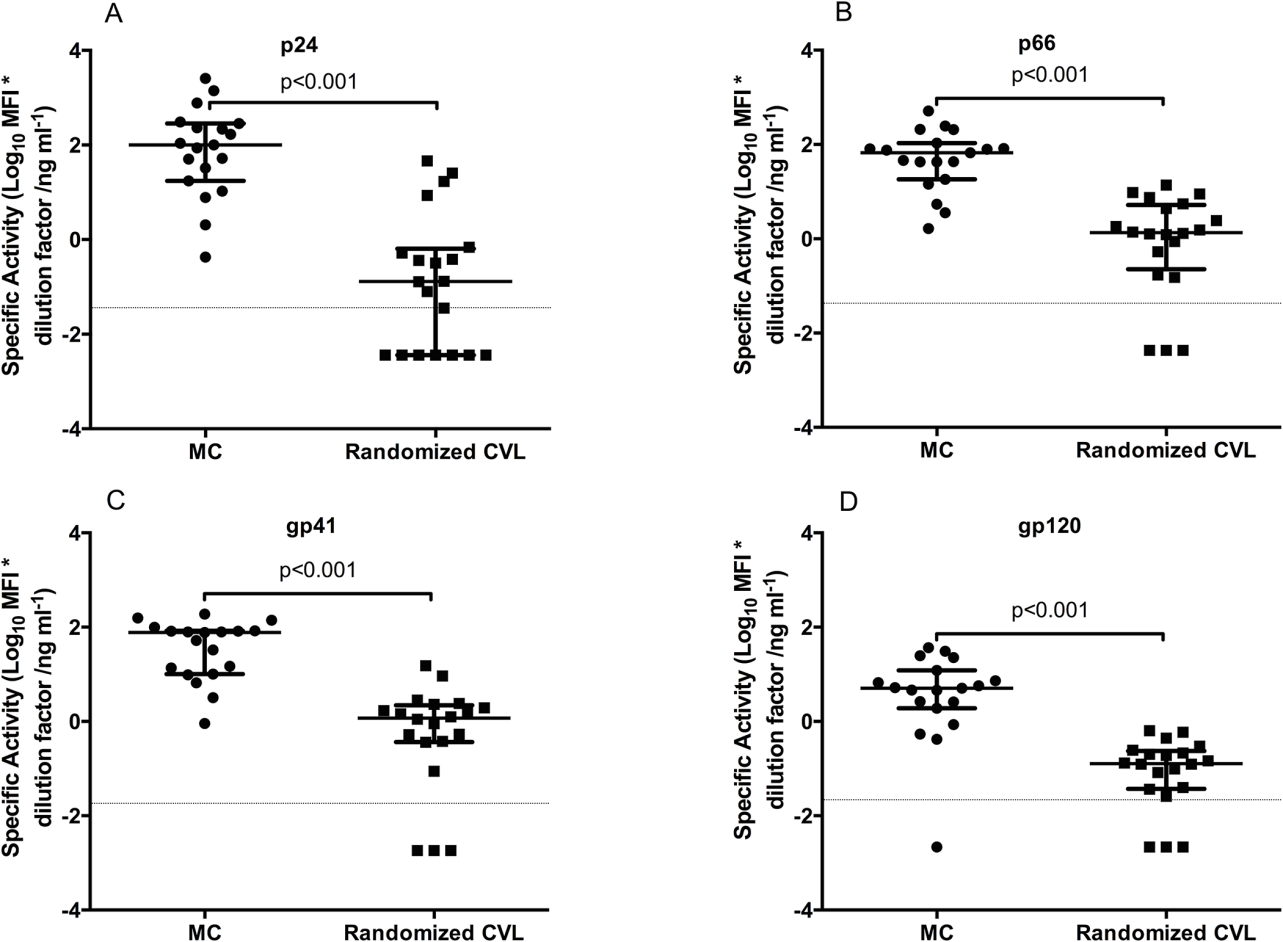
HIV specific activity [Log_10_ MFI*dilution factor ng ml^-1^ (MFI/total Ig)] for gag p24- 2A, p66- 2B, gp41- 2C and gp120- 2D in MC (n = 19) and randomized CVL samples (n = 20). Limit of detection is shown as the dotted line on the figures.

**Fig 3:**
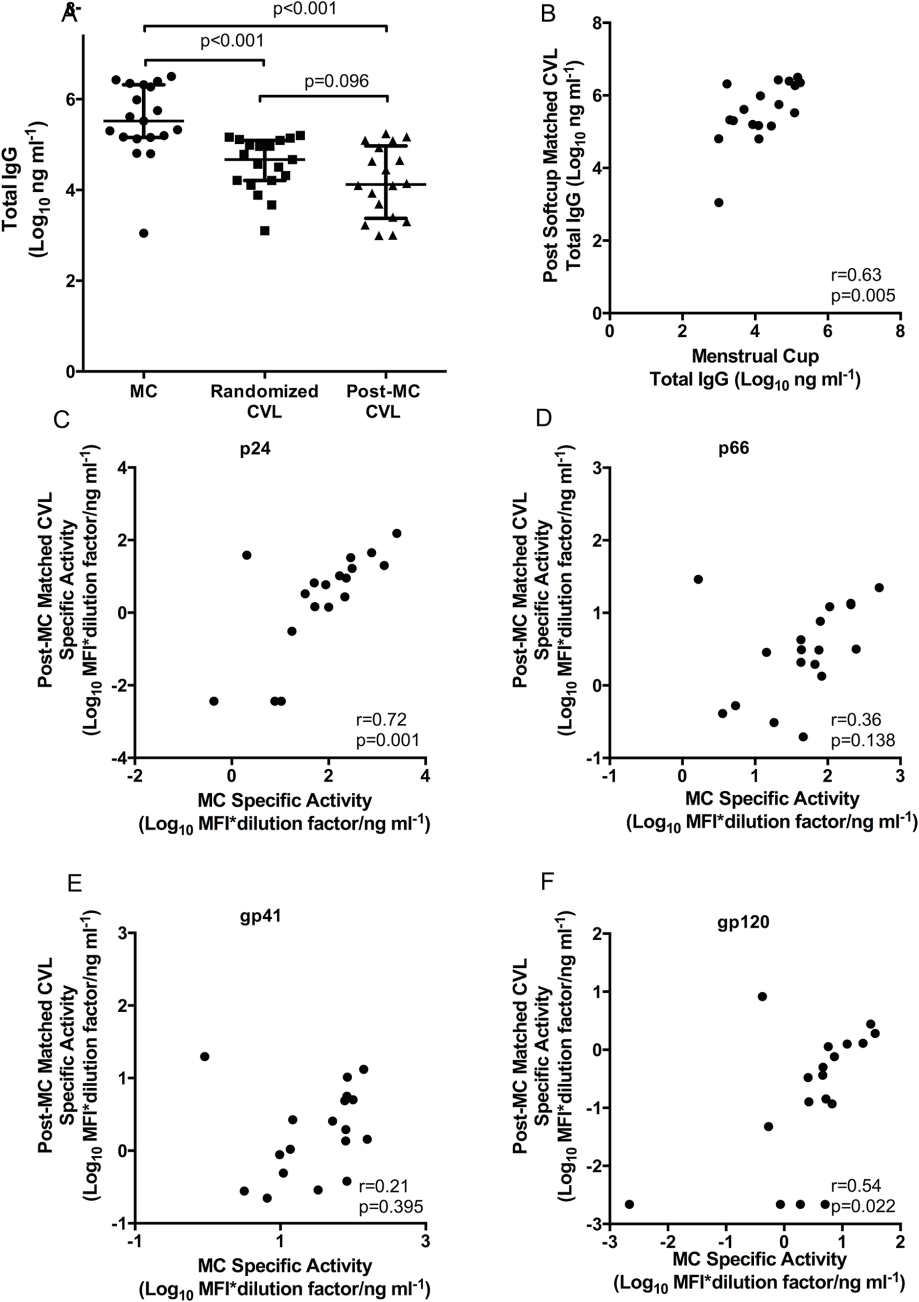
Comparison of total antibody (ng ml-1) in MCs (n = 19)- 3A, randomized CVL (n = 20) and matched post-MC CVLs (n = 18) [Log_10_ MFI*dilution factor ng ml^-1^ (MFI/total Ig)]- Correlation plots between total antibody in MCs and matched post MC CVLs- 3B. Correlation plots (Fig 3C–3F) of HIV specific activity in MCs (n = 18) and matched post-MC CVLs (n = 18) [Log_10_ MFI*dilution factor ng ml^-1^ (MFI/total Ig)] for gag p24- 3C, p66- 3D, gp41- 3E and gp120- 3F.

Specific activity was compared between MCs and randomized CVLs in order to evaluate the differences in magnitudes for the HIV-specific binding antibodies. As shown in Fig 2A–2D, levels of p24, p66, gp41 and gp120-specific activities were significantly higher (all p<0.001) in MCs than in randomized CVL samples. Total IgG levels were also higher in MCs compared to randomized CVL samples (p<0.001) (Fig 3A). In addition, post-MC CVLs were measured for total IgG and compared to those of randomized CVLs to investigate an impact on subsequent levels of IgG secreted with prior MC sampling. Interestingly, while there was a trend towards lower levels of total IgG in post-MC CVL samples, these were not significantly different to randomized CVL levels (p = 0.096), suggesting minimal impact of prior MC sampling.

All of the MC samples (19/19) had detectable responses to p24, p66 and gp41, and 95% (18/19) had detectable responses to gp120. In comparison, 85% (17/20) of randomized CVL samples had detectable responses for p66, gp41 and gp120, while only 65% (13/20) had detectable responses to p24. Overall, the mucosal IgG responses against four HIV-antigens were significantly detected in 99% of MCs compared to only 80% of randomized CVLs (p = 0.029). Within MCs, the magnitude of the HIV-specific IgG titres were similar for p24, p66(RT), and gp41 MN. Compared to all the other specificities, however, the magnitude of gp120-specific IgGs was significantly lower (p<0.001; S1A Fig).

In randomized CVLs, p24, gp41 and gp120 specific activities were significantly lower than p66 (p<0.05- S1B Fig). These results show that the MC method may be more robust allowing for detection of a similar range of magnitude across various HIV-specific IgGs compared to CVL, where dilution is likely to affect sensitivity to detect certain humoral immune specificities.

### Total and specific HIV antibodies correlate between menstrual cup and post- menstrual cup CVL

To determine whether the magnitudes of the antibodies in the MC correlated with the subsequent matched CVL samples, the total antibody between the two samples was compared. Total IgG titres correlated strongly within the same participants (r = 0.63, p = 0.005) (Fig 3B).

Of the four HIV-specificities measured in this study, MC concentrations of p24 (r = 0.72; p = 0.001), and gp120 (r = 0.54; p = 0.022) (Fig 3C and 3F) correlated significantly with matched post-MC CVL samples, while gp41 (r = 0.21; p = 0.395) and p66 (r = 0.36; p = 0.138) did not (Fig 3D and 3E).

### HIV-specific activity in post-menstrual cup CVL sampling is comparable to randomized CVL

Mucosal sampling by CVL post-MC was compared to the randomized CVL. There were no significant differences in total IgG (p = 0.096) (Fig 3A) and HIV specific activity for gp41 (p = 0.211), gp120 (p = 0.132) and p66 (p = 0.090), while p24 (p = 0.019) was significantly higher in the post-MC CVL compared to the randomized CVL sample (S2A–S2D Fig).

### Genital cytokines are more frequently detected in Menstrual Cup fluid than in randomized CVL specimens

The detectability of 48 cytokines were compared in genital secretions from women randomized to the MC (n = 19) versus CVL arms (n = 20; Table 2). Cytokines were more frequently detected in MC specimens (42/48 cytokines observed above LLOD) compared to CVL (26/48 cytokines above LLOD; p<0.001). Twenty-five of the 48 cytokines assessed were consistently observed above the LLOD in both MC and CVL. Of the remaining 23, one, IL-12p40, was detected in 95% MC specimens versus 100% CVL specimens; and 17 were observed in 100% of MC specimens, but not CVL specimens: IL-4, IL-13, TNF-α, 35%; IL-5, 45%; IL-2, G-CSF, IFN-γ 50%; PDGF-ββ, 60%; RANTES, 70%; IL-7, IP-10, 75%; IL-9, 80%; MIP-1α, 90%; and SCF, IL-12p70, Il-17, IL-8, in 95% CVL specimens (Table 2). Eleven of these 23 cytokines remained statistically different between sampling methods after multiple comparisons adjustment, all detected more frequently in MC specimens than CVL. Cytokines with concentrations below the LLOD in ≥40% of participants were classified as categorical variables for analyses of detectable concentrations. This occurred in 1/48 (2.1%) cytokines assessed in MC specimens compared to 11/48 (22.9%) cytokines assessed in CVL specimens (p = 0.004).

**Table 2:** Comparison of cytokine concentrations and detection frequencies in genital fluid isolated from menstrual cups and CVLs in a randomised study.

	Menstrual Cup (n = 19)			Randomized CVL (n = 20)					
Analyte	Median(pg/ml)	IQR(pg/ml)	≤LLOD^(%)^	Median(pg/ml)	IQR(pg/ml)	≤LLOD^(%)^	Fold difference	p-value^c^	p-value^d^
Eotaxin	17.5	5.4–30.8	15.8	0.2	0.2–0.2	90.0	85.1	<0.001^§^	<0.001^§^
IL-13	3.1	1.9–6.5	0.0	0.1	0.1–0.8	65.0	36.0	<0.001^§^	<0.001^§^
IL-4	1.6	0.9–2.0	0.0	0.1	0.1–0.6	65.0	20.7	<0.001^§^	<0.001^§^
IFN-γ	67.6	37.6–176.7	0.0	3.5	1.8–22.1	50.0	19.3	<0.001^§^	<0.001^§^
PDGF-ββ	42.7	32.4–79.7	0.0	2.9	0.2–17.3	40.0	14.6	<0.001^§^	0.003^§^
MCP-3	122.1	80.1–166.5	5.3	8.7	8.7–29.8	60.0	14.1	<0.001^§^	<0.001^§^
IL-7	5.0	3.1–11.1	0.0	0.7	0.1–1.9	25.0	7.6	<0.001^§^	0.047
IL-9	7.3	3.7–10.1	0.0	1.2	0.8–4.5	20.0	5.9	<0.001^§^	0.106
TNF-β	3.5	2.2–4.8	0.0	1.5	0.8–1.7	0.0	2.3	<0.001^§^	N/A
IFN-α2	65.1	55.4–82.3	0.0	35.1	24.1–48.9	0.0	1.9	<0.001^§^	N/A
TNF-α	26.6	15.6–86.6	0.0	0.6	0.6–9.7	65.0	42.5	<0.001^§^	<0.001^§^
HGF	803.5	381.3–1986.1	0.0	31.0	24.0–355.2	0.0	26.0	<0.001^§^	N/A
IL-6	15.1	5.3–51.7	0.0	0.8	0.5–4.7	0.0	19.6	<0.001^§^	N/A
M-CSF	575.0	280.6–997.8	0.0	102.4	29.9–242.9	0.0	5.6	<0.001^§^	N/A
SDF-1α	340.9	174.1–606.9	0.0	81.6	51.1–126.2	0.0	4.2	<0.001^§^	N/A
IL-17	62.1	39.6–114.4	0.0	18.6	12.1–32.5	5.0	3.3	<0.001^§^	1.000
IL-16	86.0	56.1–116.3	0.0	33.3	12.2–49.7	0.0	2.6	<0.001^§^	N/A
IL-2Rα	78.3	44.4–102.7	0.0	35.1	23.6–56.4	0.0	2.2	<0.001^§^	N/A
IL-5	0.6	0.4–3.5	0.0	0.0	0.0–0.6	55.0	6300	0.002^§^	<0.001^§^
G-CSF	1225.4	280.4–3678.4	0.0	18.9	1.7–441.9	50.0	64.8	0.002^§^	<0.001^§^
IL-2	4.6	2.1–15.0	0.0	0.8	0.0–3.2	50.0	6.0	0.002^§^	<0.001^§^
b-NGF	1.4	0.6–2.2	5.3	0.4	0.2–0.9	10.0	3.6	0.003^§^	1.000
MCP-1	38.8	22.2–99.9	0.0	7.1	6.1–15.3	0.0	5.4	0.003^§^	N/A
IL-8	966.5	533.8–7947.1	0.0	41.5	9.9–671.5	5.0	23.3	0.004^§^	1.000
SCF	22.7	15.1–73.4	0.0	8.8	3.9–21.0	5.0	2.6	0.004^§^	1.000
VEGF	1255.6	689.3–3578.2	0.0	371.6	149.5–1020.1	0.0	3.4	0.004^§^	N/A
LIF	42.0	25.5–55.0	0.0	15.7	10.9–26.0	0.0	2.7	0.004^§^	N/A
FGF basic	36.7	24.1–43.6	0.0	19.9	14.3–24.1	0.0	1.8	0.004^§^	N/A
IL-18	1967.0	406.2–3172.1	0.0	102.9	17.0–1352.7	0.0	19.1	0.005^§^	N/A
MIP-1α	1.9	1.5–4.1	0.0	0.7	0.4–2.2	10.0	2.8	0.006^§^	0.487
MIG	46850.	2823.7–19683.5	0.0	1275.8	111.0–4158.4	0.0	3.7	0.006^§^	N/A
IL-3	164.9	99.1–223.7	0.0	101.4	73.4–117.9	0.0	1.6	0.006^§^	N/A
IP-10	1342.5	119.0–10423.2	0.0	94.8	6.3–742.7	25.0	14.2	0.009^§^	0.047
GROα	464.7	314.4–730.6	0.0	43.8	35.3–485.7	0.0	10.6	0.009^§^	N/A
IL-1β	393.9	57.7–1754.9	0.0	18.5	5.0–274.6	0.0	21.3	0.013^§^	N/A
IL-12p70	90.0	22.8–130.2	0.0	36.6	9.3–75.0	5.0	2.5	0.017^§^	1.000
MIP-1β	12.1	8.0–55.9	0.0	4.9	2.6–16.8	0.0	2.5	0.021^§^	N/A
IL-1α	276.7	192.6–1000.5	0.0	155.8	32.8–404.5	0.0	1.8	0.023^§^	N/A
RANTES	13.5	5.2–22.3	0.0	5.6	0.4–9.0	30.0	2.4	0.024^§^	0.020^§^
TRAIL	77.9	48.7–235.8	0.0	20.1	8.3–122.9	0.0	3.9	0.026^§^	N/A
IL-12p40	194.8	96.0–423.7	5.3	360.1	318.3–441.1	0.0	0.5	0.031^§^	0.487
SCGF-β	19.8	19.8–68.1	68.4	19.8	19.8–19.8	95.0	1.0	0.039^§^	0.044
GM-CSF	38.3	28.9–118.2	0.0	107.1	96.4–112.8	0.0	0.4	0.046	N/A
IL-15	1.5	0.3–5.0	21.1	6.4	3.1–11.9	20.0	0.2	0.057	1.000
IL-1rα	22096.4	1594.3–33497.0	0.0	5673.2	1592.7–13686.3	0.0	3.9	0.069	N/A
IL-10	19.0	3.3–31.6	0.0	11.1	7.2–20.0	0.0	1.7	0.405	N/A
CTACK	48.3	33.7–62.7	0.0	42.8	33.4–49.6	0.0	1.1	0.452	N/A
MIF	1601.9	354.1–5255.9	0.0	361.0	60.2–11069.8	0.0	4.4	0.802	N/A

^a^IQR: Interquartile range.

^b^LLOD: Lower limit of detection.

^c^Mann-Whitney U tests were conducted to compare non-parametric unpaired data.

^d^Fisher’s exact tests were conducted to compare detection frequencies.

§Significant P-value after FDR multiple comparison adjustment.

### Menstrual cup specimens yield greater cytokine concentrations

With the exception of IL-15, IL-10, CTACK, MIF, IL-1Rα, and IL-12p40, the median concentrations of 41/48 cytokines were significantly higher in MC than randomized CVL specimens (Table 2). Only one of these 41 cytokines, GM-CSF, did not maintain statistical significance after FDR adjustment. IL-12p40 concentrations were greater in the randomized CVL than menstrual cup samples (median 360.1pg/ml in CVL vs 194.8 in MC, p = 0.031) while IL-15, IL-10, CTACK, MIF, and IL-1Rα were not different between samples.

### Menstrual cup sampling does not alter the cytokine milieu

**Fig 4:**
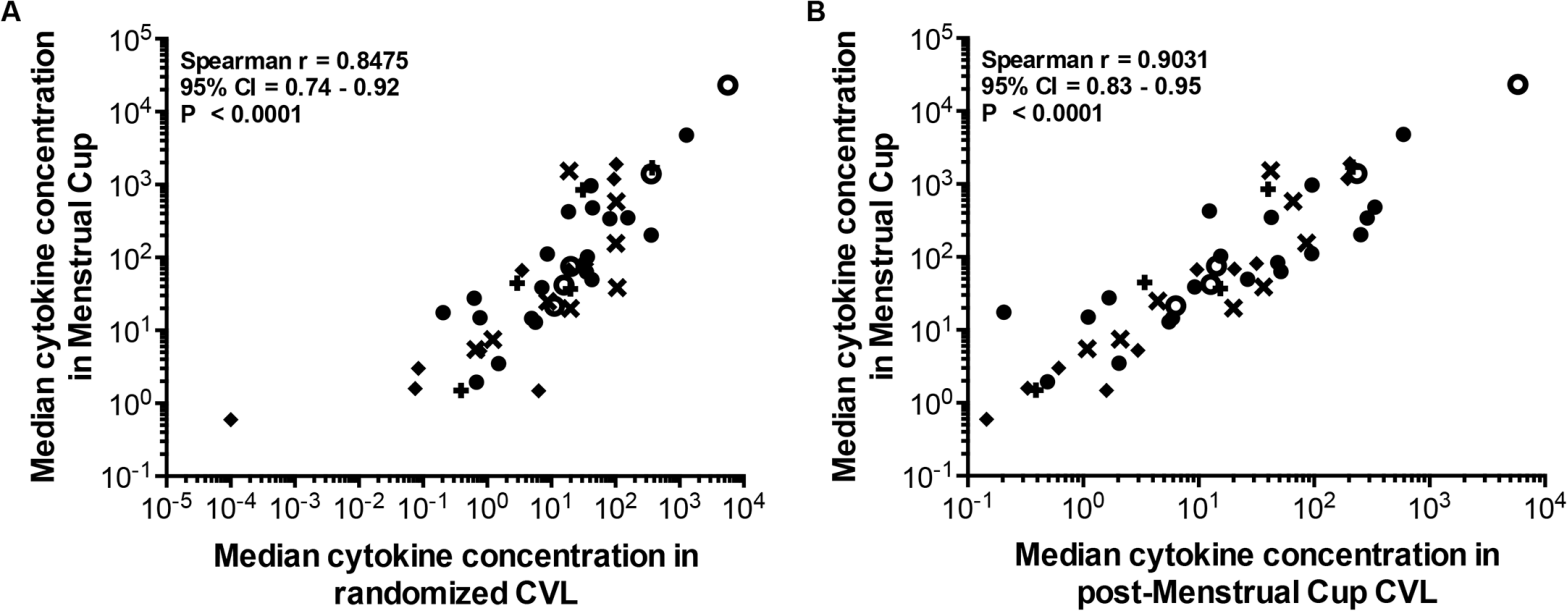
Comparison of median cytokine concentrations observed in genital fluid isolated from CVL, MC and matching subsequent CVL specimens in a randomised study. Cytokine concentrations were compared in genital fluid isolated from n = 19 MC- 4A and n = 20 randomized CVL, and n = 18 matching MC and subsequently-sampled CVL specimens- 4B. The concentrations of 48 cytokines [(regulatory ○; growth factor ✚; haematopoietic ✖; adaptive ◆; inflammatory ●)] were determined in each specimen by luminex technology and the median of each cytokine concentration was compared by Spearman rank correlation. Data are depicted on Log_10_ scales. P-values <0.05 were considered significant.

The median cytokine concentrations in randomised MC specimens were similarly ranked to that isolated from randomised CVL specimens (Spearman r = 0.85, 95% CI 0.74–0.92; p<0.001; Fig 4A), indicating a similar profile of cytokines isolated from MC and CVL specimens. This trend was also observed when comparing the median cytokine concentrations in CVL sampled immediately after MC removal (n = 18) with that of matching MC specimens (Spearman r = 0.903, 95% CI 0.83–0.95, p<0.001; Fig 4B). Furthermore, comparing cytokine concentrations in CVL collected after MC sampling (n = 18) with CVLs collected from women randomised to undergo CVL sampling alone (n = 20), 47/48 cytokines measured were similar in concentration, further supporting the observation that CVL sampling immediately after MC removal does not significantly alter the cytokine profile. Only CTACK concentrations differed between groups (p<0.001), being 38% lower in CVL sampled after MC (median 26.6 pg/ml, IQR 14.5–34.4 pg/ml) compared to CVL sampled alone (42.8 pg/ml, IQR 31.7–50.2 pg/ml).

## Conclusions

This first randomized study comparing MC and CVL mucosal sampling has shown that MC sampling is a superior and preferred method for the collection of immune markers than the conventional CVL sampling. MC sampling consistently allowed for more frequent detection and reliable quantification of HIV-specific IgGs and cytokines, demonstrating the superior sensitivity of this method over CVL sampling, in particular for markers present at low concentrations. In addition, this study also demonstrated that MC insertion did not significantly impact the immune environment when the subsequent CVL was analysed.

Further benefits of MC sampling extend from the acquisition of concentrated genital fluid where the precise dilution can be calculated from the original fluid. The neat sample obtained from MC can be diluted substantially more than CVLs for the detection of humoral responses in particular, and these dilutions can be modified for the different sensitivities for various assays and to generate consistent data.

While this study provided a substantial description of the HIV-specific antibody and genital cytokine milieu detectable in genital secretions isolated from MC and CVL, the experimental assays conducted were not exhaustive. Further investigation into sampling-associated differences using additional assays could verify the impact of MC sampling on the genital cellular environment, epithelial barrier integrity, broader antibody profile and other factors. These would also facilitate interpretation of isolated differences between sampling approaches such as the reduction in both MC IL-12p40 and post-MC CVL CTACK concentrations relative to randomised CVL specimens.

Multiple studies have compared humoral and innate immune markers using various collection methods including Weck-Cel or Merocel sponges, swabs and ophthalmic sponges, and CVL. These methods were found to yield differing results for the detection and concentrations of immune markers [5,24,25]; although Merocel sponges were found to yield superior quality and quantity of immune markers than Dacron or flocked nylon swabs [11], other studies have shown that flocked swabs were superior to Dacron swabs for detection of STIs [26,27]. In addition, variability in the detection of immune markers can be introduced by sampling different areas of the genital tract [28], or through inter-individual variability [2,4,8]. Concern that MC sampling may include secretions from the cervical os and surrounding vaginal walls that may be absent from CVL is mitigated by this study’s observation of similar profiles of both antibodies and cytokines in MC and CVL. Furthermore, while the drawback for most of these methods is the reliance on healthcare professionals, MCs can be self-inserted and removed with appropriate training [12,13]. The MC circumvents the disadvantages of the more common swab or lavage methods, including the need for speculum insertion, dilution [8] and inconsistent genital fluid recovery [5,29]. In this study participants had some reservations to self-insert or remove the MC. However, considering the high acceptability of wearing the device and the commercial availability as a self-insertion device, the study team believe that with additional counselling and education, including the use of short videos on insertion and removal of the MC, this method could quickly become more user-friendly. Studies have reported that with training, self-sampling using MCs or other devices can be used successfully [6,12–14]. A single study by Boskey et al. (2003) with n = 16 participants in the USA, showed that a 5 second self-insertion of the MC yielded an average of 0.5g of cervicovaginal fluid secretions [13]. Beyond this study a wide range of expert opinions exist recommending anything from insertion with immediate removal to overnight placement [13,17]. In addition, other factors like hormonal contraceptive use were not reported in the Boskey study. Hormonal contraception (56% in our study) is an important factor that could affect the quality [30] and yield of genital secretions. The rationale for the insertion of the MC for two hours therefore was a compromise between taking into account expert opinions and practicality of participant follow-up. A shorter sampling time with the MC may be beneficial to maintaining mucosal sample integrity by reducing the time of exposure of the vaginal vault, which is a hypoxic environment, to the MC. MC would be an ideal tool to collect undiluted vaginal secretions [15] in future vaccine trials and HIV prevention research to better measure and define mucosal correlates of immune protection that have thus far been described mainly in the highly exposed uninfected populations [31–39], an area that remains a significant gap in HIV research.

A limitation of this study was that menstrual history data was not available for all participants, and therefore prevented the assessment of the role of the menstrual cycle on the volume and composition of genital fluid. However, the random allocation of women to the sampling methods would have minimized a possible selection bias. Another limitation is that total volume of genital secretions recovered from the MC was not measured, however, post-collection weighing of the secretions in the MC is a suitable alternative [13]. A possible caveat to this method is that the weights of the MCs themselves could vary between the batches which may over- or under-estimate the weight of the secretions and may likely incur logistical problems in large-scale studies. In addition, while antibody measurements were adjusted by total IgG in both specimens, no similar method of standardisation, for example, lithium chloride [9,40] or the bicinchoninic acid adjustments [3], was conducted for cytokine evaluations. This would have been particularly useful in CVL specimens where absolute dilution factor is not known. However, to standardize the cytokine concentrations observed in MC and CVL, specimens were not adjusted for dilution factors since the dilution of genital fluid in saline as collected by CVL sampling was not known. Despite this, the cytokine multiplex assays allowed the quantification of a diverse panel of soluble immune markers that mirrored the trends observed in the normalised antibody assays.

In conclusion, MC collection represents a convenient and more practical alternative to other genital sample collection methods such as CVL, and may circumvent the need for healthcare professional driven genital sampling. MC is a robust tool for the detection and quantification for humoral and soluble biomarkers and can be used to more accurately study immune responses in genital secretions. It will be important to assess their utility for genital mucosal sampling in vaccine trials to identify immune correlates of protection. If warranted and given the practicality, MC could be widely implemented in the field.

## Supporting Information

S1 Fig Intragroup comparison of magnitude of HIV specific activity in MCs (n = 19)- 1A and randomized CVLs (n = 20)- 1B [Log_10_ MFI ng ml^-1^ (MFI/total Ig)] for—gag p24, p66, gp41 and gp120. (TIFF)

S2 Fig HIV specific activity in post-MC CVLs (n = 18) and randomized CVLs (n = 20) [Log_10_ MFI*dilution factor ng ml^-1^ (MFI/total Ig)] for gag p24- 2A, p66- 2B, gp41- 2C and gp120- 2D. Limit of detection is shown as the dotted line on the figures.
(TIFF)

S1 File Data for PlosOne_anonymized- 02 June 2015.pdf. (PDF)
