# Supplementary figures and images for: Randomized Cross-Sectional Study to Compare HIV-1 Specific Antibody and Cytokine Concentrations in Female Genital Secretions Obtained by Menstrual Cup and Cervicovaginal Lavage
Source: PLoS One. 2015 Jul 6;10(7):e0131906. doi: 10.1371/journal.pone.0131906 (PMC4492781; doi:10.1371/journal.pone.0131906)

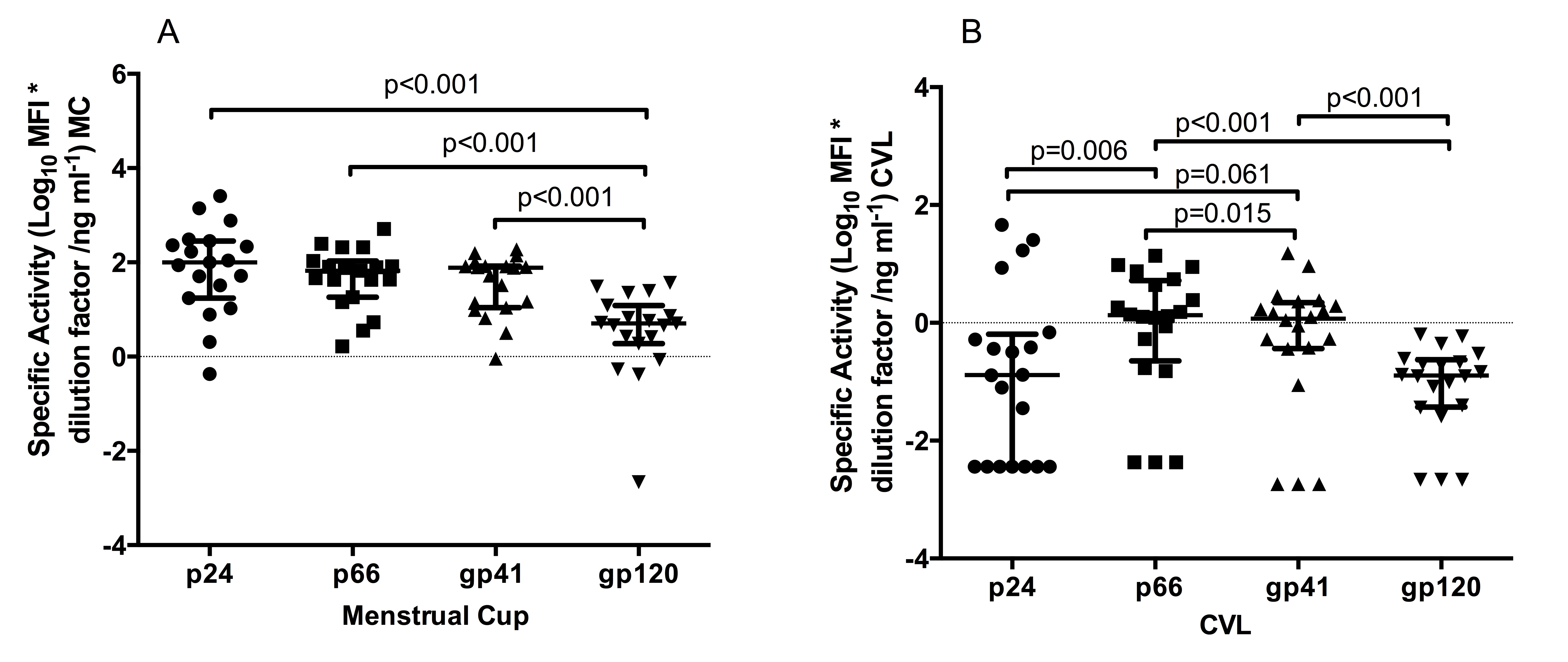

Supplement: S1 Fig — (TIFF) [file pone.0131906.s001.tiff]

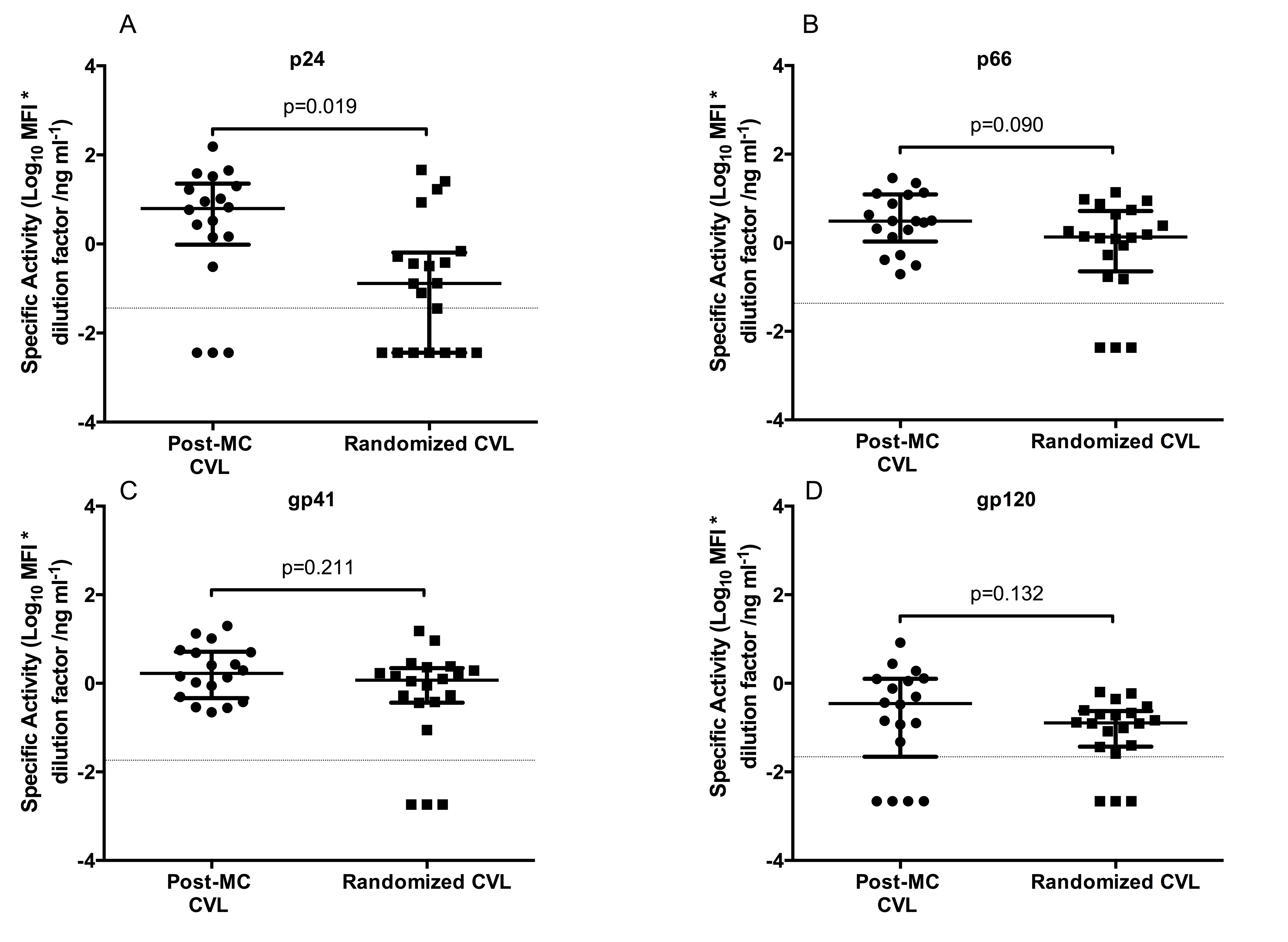

Supplement: S2 Fig — Limit of detection is shown as the dotted line on the figures. (TIFF) [file pone.0131906.s002.tiff]
