# Supplementary material for: Randomized Cross-Sectional Study to Compare HIV-1 Specific Antibody and Cytokine Concentrations in Female Genital Secretions Obtained by Menstrual Cup and Cervicovaginal Lavage
Source: PLoS One. 2015 Jul 6;10(7):e0131906. doi: 10.1371/journal.pone.0131906 (PMC4492781; doi:10.1371/journal.pone.0131906)
Supplement: S1 File — (PDF) [file pone.0131906.s003.pdf]

PID	Rando	p24_rawSC	p66RT_rawSC	gp41_rawSC	gp120_rawSC	TotalIGG_SC	p24_usedSC
0001	*						
0002	*						
0003	*						
0004	*						
0005		130695	105645	57997,5	26895	64001,75	2,04
0006	*						
0007		13755625	16840375	22081625	4859125	158051,34	87,03
0008		966560000	661185000	242900000	14545875	3165662,05	305,33
0009		117387500	32502500	34406250	2356600	412234,43	284,76
0010		87200000	113943750	219571250	32371250	2669339,05	32,67
0011	*						
0012		110678750	6212500	1401900	665650	143477,22	771,4
0013		11715	15975	16425	50	1112,16	10,53
0014	*						
0015	*						
0016		96800000	124085000	147547500	42303750	1862013,15	51,99
0017	*						
0018		104583750	7409000	6616250	5360250	2077582,86	50,34
0019	*						
0020	*						
0021		209437500	239325000	151700000	6462750	970065,17	215,9
0022	*						
0023		14584750	10812875	25240000	3313125	133911,92	108,91
0024	*						
0025		85537,5	1079925	1322062,5	380737,5	200698,73	0,43
0026	*						
0027		488302,5	2899675	5184800	318862,5	62978,69	7,75
0028	*						
0029	*						
0030	*						
0031		462842500	68512500	32931250	2410000	330105,8	1402,1
0032	*						
0033		128672500	42422500	7651000	300850	559799,92	229,85
0034		376125000	75312500	7663350	5392850	147007,56	2558,54
0035		38881250	184543750	176833750	11665000	2237939,77	17,37
0036	*						
0037		416742500	105642500	27097500	6546750	2470194,75	168,71
0038		21156250	3868400	6980000	181500	211875,14	99,85
0039	*						

SC= SoftCup or Menstrual Cup Fluid
CVL= Cervicovaginal Lavage
* = Randomized CVL

p66_usedSC	gp41_usedSC	gp120_usedSC	p24_rawCVL	p66RT_rawCVL	gp41MN_rawCVL
			7496,1	3536,1	1820,1
			50	5951,1	1776,6
			50	1170	4140,9
			944525	277930	33087
1,651	0,906	0,4202	37967,1	28518,6	19535,1
			2080,5	24827,4	18003
106,55	139,712	30,744	50103,6	103503	112898
208,862	76,73	4,5949	2446450	1911500	722600
78,845	83,463	5,7167	160710	37516,5	50384,4
42,686	82,257	12,1271	142007	183150	241720
			50	50	50
43,3	9,771	4,6394	1246130	86210	24420,9
14,364	14,769	0,0022	50	2878,5	2684,4
			50	21129	20363,1
			50	110820	88385
66,64	79,241	22,7194	182860	243505	243127
			4586,4	157598	67720
3,566	3,185	2,58	11208	691,5	468
			781315	397480	257666
			47080	1100165	1134190
246,71	156,381	6,6622	37795,5	43968,9	19972,5
			30910,5	237368	234908
80,746	188,482	24,7411			
			63440	80650	155242
5,381	6,587	1,8971	50	1317	555,9
			16490,4	16865,4	11535,9
46,042	82,326	5,063	50	2466	4795,5
			50	50	50
			2464080	1392830	282680
			18282,6	192563	219873
207,547	99,76	7,3007	2421570	1645170	608920
			2139530	256574	107294
75,782	13,667	0,5374	403626	137766	47034,6
512,304	52,129	36,6842	1916200	276584	31785,9
82,461	79,016	5,2124	53995,5	234425	237632
			12586	2184350	2397050
42,767	10,97	2,6503	894350	178884	42284
18,258	32,944	0,8566	2835	615,9	576
			50	50	50

gp120C_rawCVL	TotalIGG_CVL	p24_usedCVL	p66_usedCVL	gp41_usedCVL
537,6	20772	0,361	0,1702	0,0876
888,6	4690,68	0,004	1,2687	0,3788
305,4	7725,38	0,004	0,1514	0,536
23391	36955,09	25,559	7,5208	0,8953
8134,5	986,99	38,468	28,8945	19,7926
2112	16147,32	0,129	1,5376	1,1149
23563,5	8530,15	5,874	12,1338	13,2352
53728,5	147666,91	16,567	12,9447	4,8934
5538,9	4902,2	32,783	7,653	10,2779
54052,5	42984,98	3,304	4,2608	5,6234
50	0	0,004	0,0043	0,0018
13842,9	27751,24	44,904	3,1065	0,88
50	1006,04	0,004	2,8612	2,6683
2022	16242,16	0,004	1,3009	1,2537
5010	60819,4	0,004	1,8221	1,4532
161345	124765,83	1,466	1,9517	1,9487
12730	129315,33	0,035	1,2187	0,5237
561	1685,29	6,65	0,4103	0,2777
11292	90959,1	8,59	4,3699	2,8328
37004,4	123446,55	0,381	8,9121	9,1877
1632	13901,07	2,719	3,163	1,4368
14182,5	97152,61	0,318	2,4432	2,4179
19669,5	91994,54	0,69	0,8767	1,6875
50	2506,1	0,004	0,5255	0,2218
1160,4	31801,96	0,519	0,5303	0,3627
50	12611,42	0,004	0,1955	0,3803
50	1258,5	0,004	0,0043	0,0018
29193,9	145966,37	16,881	9,5421	1,9366
82083	138898,24	0,132	1,3864	1,583
92255	121352,02	19,955	13,557	5,0178
20634,9	46676,92	45,837	5,4968	2,2987
2136,6	44913,21	8,987	3,0674	1,0472
23718,9	12450,1	153,91	22,2154	2,5531
24919,5	174857,59	0,309	1,3407	1,359
38759,4	158309,09	0,08	13,798	15,1416
10922,1	85990,01	10,401	2,0803	0,4917
50	2003,52	1,415	0,3074	0,2875
50	12672,45	0,004	0,0043	0,0018

gp120_usedCVL	IL_1b_CVL	IL_1ra_CVL	IL_2_CVL	IL_4_CVL	IL_5_CVL	IL_6_CVL	IL_7_CVL
0,02588	10,33	9898,97	5,82	0,075	0,47	0,73	0,03
0,18944	21,33	1596,81	0,04	0,075	0,0001	0,89	0,16
0,03953	4,46	2792,4	0,04	0,075	0,0001	0,65	0,03
0,63296	5,61	444,72	0,04	0,075	0,0001	0,25	0,27
8,24172	28,69	5620,28	6,5	0,075	0,0001	0,97	1,1
0,1308	8,45	10018	0,04	0,075	0,15	0,49	0,79
2,76238	8,78	8811,29	5,82	0,075	0,15	0,65	0,59
0,36385	10,21	10598,05	0,04	0,42	0,14	7,04	1,06
1,12988	31,67	2043,11	0,04	0,36	0,1	0,58	0,6
1,25747	84,12	14646,54	2,73	0,075	0,47	0,81	0,59
0,00218	0,47	84,69	0,04	0,075	0,0001	0,41	0,03
0,49882	61,09	12299,99	5,99	0,31	0,15	0,93	0,95
0,00218	2,21	163,69	0,04	0,21	0,06	0,19	1,32
0,12449	10,06	5389,29	2,13	0,075	0,0001	0,49	0,48
0,08238	1,47	2049,02	1,72	0,075	0,0001	0,81	0,27
1,29318	158,14	20811,22	8,65	0,42	0,85	102,94	2,01
0,09844	72,78	18622,34	0,04	0,62	1,48	1,54	1,86
0,33288	10,48	2731,6	6,16	0,075	0,56	1,05	0,38
0,12414	712,94	11802,32	8,08	1,31	0,94	31,53	10,42
0,29976	2,56	1588,6	4,5	0,075	0,0001	0,49	0,03
0,1174	9,47	628,45	4,48	1,93	0,41	1,2	1,52
0,14598	113,1	32918,16	4,5	1,47	2,07	60,55	6,89
0,21381	15,74	5957,02	0,04	0,075	0,0001	0,65	0,79
0,00218	27,77	605,39	0,04	0,33	0,06	1,16	0,39
0,03649	412,67	14561,51	3,02	0,67	1,21	6,48	1,91
0,00218	5,29	10985,31	4,05	0,075	0,0001	1,18	1,15
0,00218	28,29	175,8	1,51	0,075	0,0001	0,41	0,03
0,2	1,3	171,98	0,04	0,075	0,0001	0,73	0,64
0,59096	1510,69	20034,8	3,21	0,71	0,26	9,76	1,91
0,76023	94,85	25543,73	0,23	0,76	0,17	3,94	2,32
0,44208	136,5	3285,5	0,04	0,075	0,0001	3,56	0,69
0,04757	0,17	651,87	0,08	0,55	0,0001	0,32	0,17
1,90512	14,34	6032,1	0,04	0,33	0,1	1,3	1,32
0,14251	734,71	4593,55	3,22	1,43	0,24	60,27	2,86
0,24483	623,96	24992,72	0,04	0,46	0,76	2,34	3,05
0,12702	6,32	25631,86	0,04	0,76	0,14	3,42	1,82
0,00218	0,41	1421,82	6,24	0,075	0,26	0,49	0,22
0,00218	479,52	12811,05	3,21	0,5	0,15	5,75	1

IL_8_CVL	IL_9_CVL	IL_10_CVL	IL_12p70_CVL	IL_13_CVL	IL_15_CVL	IL_17_CVL	Eotaxin_CVL
9,07	0,74	14,66	9,33	0,085	16,53	21,31	0,205
4,19	0,89	6,42	16,58	0,085	6,35	14,12	0,205
24,41	0,27	8,14	2,14	0,085	12,23	12,75	0,205
62,7	0,27	3,74	9,33	0,085	8,97	17,74	0,205
33,39	1,91	12,09	13,51	0,085	17,86	18,64	0,205
10,8	1,28	13,7	28,1	0,085	0,0001	11,37	0,205
20,97	2,14	15,31	43,1	0,085	12,79	21,31	0,205
261,08	1,47	2,77	7,42	2,21	0,08	4,42	2,44
136,15	0,54	1,77	5,63	0,55	0,52	3,17	0,41
67,85	2,14	14,82	20,17	0,68	8,18	25,72	0,205
0,22	0,27	3,06	0,275	0,085	0,0001	10,44	0,205
72,84	2,99	15,63	36,73	0,085	17,51	22,2	0,205
10,08	1,2	4,4	46,49	3,55	0,56	3,37	0,205
122,2	1,09	10,82	24,05	0,085	11,48	13,21	0,205
3,84	1,18	8,76	64,12	0,085	3,37	0,165	0,205
160,75	3,5	20,03	47,23	0,8	17,55	58,49	12,15
19,76	2,43	18,88	45,16	0,68	3,64	23,08	0,205
300,22	2,9	8,84	12,34	0,085	19,57	26,16	0,205
12569,29	10,4	28,29	100,14	4,63	9,41	73,78	7,95
12,87	0,27	9,55	76,89	0,085	15,86	9,51	0,205
1145,86	5,93	2,31	7,6	0,78	1,62	117,61	34,17
3793,29	8,62	27,79	87,99	4,63	2,79	47,18	0,205
56,25	1,09	11,46	69,38	0,085	4,55	10,44	0,205
26,76	1,01	1,33	3,26	0,31	0,31	5,3	0,205
253,46	4,65	21,26	46,19	0,8	3,64	29,22	0,205
32,7	3,36	12,49	9,1	0,085	16,25	19,53	0,205
26,65	1,09	3,96	2,47	0,085	16,06	15,94	0,205
3,75	1,48	3,36	9,33	0,085	15,78	70,08	6,24
1234,77	5,02	20,85	75,93	0,8	0,0001	53,9	0,205
118,83	3,73	7,27	70,53	4,29	0,41	8,29	8,08
252,59	1,48	7,98	9,67	0,085	6,35	19,53	0,205
1,42	1,6	0,93	2,37	0,085	1,57	83,41	22,23
144,92	1,2	3,38	17,5	2,12	0,95	3,57	0,205
7101,37	3,26	5,45	44,75	3,14	1,44	56,55	30,79
8190,34	11,06	30,3	159,92	2,05	0,0001	35,7	0,205
690,29	2,89	3,89	23,8	2,34	0,84	9,96	4,36
1,17	0,27	7,2	3,02	0,085	19,27	24,85	0,205
1089,47	4,38	19,05	74,15	0,74	10,21	27,91	0,205

FGFbasic_CVL	G_CSF_CVL	GM_CSF_CVL	IFN_g_CVL	IP_10_CVL	MCP_1_CVL	MIP_1a_CVL
15,2	36,12	106,48	1,815	0,42	7,14	0,62
10,07	1,71	101,49	1,815	0,42	6,74	0,62
13,53	1,71	107,33	1,815	13,61	5,82	0,45
24,55	45,59	106,22	1,815	389,57	5,3	0,33
15,61	52,84	106,13	6,6	102,22	11,1	0,87
13,09	1,71	106,82	7,96	17,41	6,13	0,27
14,79	51,05	117,3	1,815	129,43	8,5	0,49
6,36	80,99	24,33	21,11	635,53	12,99	0,33
8,04	5,62	30,65	11,61	176,47	4,54	0,24
21,19	1,71	117,81	5,91	213,31	13,83	1,12
11,51	1,71	92,96	1,815	0,42	4,87	0,22
19,42	105,45	125,88	6,6	826,36	6,64	0,97
3,29	7,8	30,65	3,63	18,38	5,75	0,14
18,32	1,71	110,26	1,815	1094,63	6,64	0,41
17,94	1,71	119,02	5,2	0,42	6,03	0,005
19,42	2772,15	125,8	25,47	2031,7	81,93	1,87
19,05	66,74	95,29	15,56	35,22	7,68	1,3
25,2	1,71	117,64	5,91	800,35	10	0,59
45,81	18500,55	111,37	55,54	8565,57	40,4	31,98
15	1,71	126,65	1,815	154,29	7,09	0,005
64,24	32,88	36,48	130,44	99,98	6,37	1,86
28,92	2778	114,72	34,82	4004,09	35,95	2,11
13,09	1,71	94,25	1,815	313,63	7,14	0,49
8,04	28,78	30,99	7,69	99,18	4,68	0,22
20,84	498,13	97,01	21,47	23,43	7,73	3,34
19,78	1,71	120,9	1,815	132,21	49,23	0,33
21,54	1,71	109,74	1,815	0,42	5,61	0,74
29,22	1,71	117,56	1,815	12,12	24,16	0,77
32,57	3892,2	94,77	48,45	5670	21,21	2,42
12,87	553,73	36,14	26,67	1162,5	4,97	0,4
23,57	425,27	95,81	9,28	279,82	15,57	1,47
36,82	6,71	35,69	11,61	48,33	4,09	0,94
4,94	64,13	32,89	11,61	336,94	12,96	0,2
35,33	615,28	31,54	100,19	3477,27	15,37	1,58
23,57	182,58	109,48	26,03	5967,89	15,11	2,26
9,58	128,91	36,48	26,67	3722,08	12,17	0,29
15,2	1,71	120,9	5,91	57,91	8,31	0,49
23,4	458,59	114,21	22,63	390,78	12,74	2,69

PDGF_bb_CVL	MIP_1b_CVL	RANTES_CVL	TNF_a_CVL	VEGF_CVL	IL_1a_CVL	IL_2Ra_CVL
2,03	3,25	6,57	0,625	122,67	326,15	21,2
3,84	3,86	0,425	0,625	201,83	485,21	39,55
0,22	2,23	5,74	0,625	25,61	17,94	43,87
4,48	5,57	0,425	0,625	141,82	28,51	21,2
3,84	5,37	5,74	0,625	139,69	121,87	25,99
0,22	7,87	0,425	0,625	302,89	444,53	25,99
0,22	3	7,15	0,625	444,78	193,66	21,2
2,33	19,71	6,39	2,98	136,57	13,1	35,62
2,97	4,54	0,425	2,45	137,26	19,72	17,23
5,74	11,99	0,425	0,625	265,72	571,12	52,3
0,22	1,32	0,425	0,625	10,74	1,38	25,99
2,53	14,32	5,3	0,625	410	42,33	16,2
2,97	3,64	0,425	1,59	881,39	31,63	30,77
14,12	4,37	4,58	0,625	259,24	53,22	21,2
0,22	1,85	3,17	0,625	780,5	26,44	56,42
4,32	45,99	16,32	0,625	502,77	137,63	99,02
2,03	5,47	5,53	5,79	661,4	249,21	25,99
0,22	4,96	7,7	0,625	167,6	10,62	21,2
20,43	446,37	35,89	38,03	1694,62	364,52	56,42
0,22	1,41	4,58	0,625	1105,25	37,13	48,12
7,51	7,58	5,18	9,14	68,9	42,76	21,09
26,73	59,48	27,52	14,06	1617,74	155,34	72,42
0,22	2,93	0,425	0,625	934,88	85,14	16,2
1,06	26,59	0,425	1,76	84,85	6,59	23,02
12,37	10,52	8,39	12,96	440,29	156,16	48,12
6,05	5,67	7,34	0,625	94,07	71,54	25,99
0,22	3,74	6,57	0,625	29,75	39,11	21,2
0,22	1,85	8,22	0,625	187,83	8,76	30,62
35,92	37,67	79,26	14,61	1864,11	1868,72	87,8
10,14	81,59	0,425	2,63	1275,07	51,03	32,71
4,48	12,71	0,425	0,625	157,12	531,37	30,62
1,53	2,81	2,72	2,98	73,29	10,66	38,54
8,17	5,72	0,425	2,63	285,55	86,1	42,43
19,77	6,17	7,78	12,01	3052,8	863,42	83,48
28,8	20,86	16,32	10,21	6408,26	1863,92	62,5
8,82	11,67	10,87	2,27	430,23	15,14	59,98
0,22	2,73	8,05	0,625	27,43	5,43	35,14
27,15	26,21	9,68	9,11	820,96	196,46	62,5

IL_3_CVL	IL_12p40_CVL	IL_16_CVL	IL_18_CVL	CTACK_CVL	GROa_CVL	HGF_CVL
62,37	270,44	12,24	14,63	30,05	24,76	12,97
120,04	446,89	46,96	1078,78	48,29	50,7	81,19
115,79	435,36	46,96	1240,17	45,6	51,32	32,1
67,96	324,86	3,54	3,41	30,05	37,55	12,97
73,35	381,8	26,96	320,05	30,05	115,41	45,44
93,32	160,92	37,76	628,91	42,81	36,35	29,82
73,35	356,97	17,98	190,01	30,05	41,69	17,99
95,09	89,19	75,58	89,04	14,52	381,57	153,12
88,31	319,8	27,1	113,85	17,41	502,05	26,85
107,06	502,79	48,39	1397,26	36,81	50,7	93,36
73,35	324,86	3,54	3,19	36,81	24,76	9,01
73,35	337,86	12,24	217,56	22,03	35,95	36,61
114,91	89,19	66,08	1054,65	14,52	323,22	43,48
67,96	284,45	12,24	19,37	30,05	34,29	25,18
115,79	387,9	17,98	19,73	87,97	28,88	45,44
191,31	627,7	105,32	4729	58,14	88,85	299,88
73,35	241,43	3,54	91,71	36,81	37,15	27,51
83,61	324,86	12,24	7,36	36,81	3277,59	15,51
107,06	350,65	162,72	50,28	55,79	6588,12	3062,17
102,58	435,36	37,76	3,19	42,81	43,47	29,82
62,95	82,45	44,01	110,53	11,43	524,28	26,85
126,29	607,64	78,72	1620,42	55,79	11261,77	181,14
78,56	311,64	12,24	114,05	26,25	39,09	22,82
54,08	54,06	48,67	11	8,04	331,71	28,79
115,79	405,97	46,96	1572,54	48,29	58,89	289,72
83,61	363,24	3,54	82,3	33,55	139,15	10,36
78,56	363,24	3,54	33,64	30,05	24,76	27,51
100,3	356,97	26,96	10,49	36,81	44,16	20,42
172,12	789,18	104,4	3829,46	60,42	838,86	5116,38
125,21	118,02	61,88	855,96	13,77	363,11	169,45
73,35	337,86	28,93	134,18	36,81	515,04	501,23
70,17	70,71	64,69	12,79	16,7	341,22	14,95
140,37	155,7	70,2	697,34	26,58	393,99	81,77
216,79	186,78	93,54	2153	32,53	669,1	817,94
128,34	513,66	52,53	1465,16	50,87	865,04	420,6
160,1	155,7	75,58	2186,04	26,58	449,8	95,06
78,56	350,65	20,45	6,14	36,81	26,88	10,36
155,67	657,33	82,95	2502,28	42,81	456,45	771,2

IFN_a2_CVL	LIF_CVL	MCP_3_CVL	M_CSF_CVL	MIF_CVL	MIG_CVL	b_NGF_CVL	SCF_CVL
18,19	15,68	8,67	75,51	68,28	38,75	0,03	0,045
48,2	22,07	29,78	323,01	12564,35	67,23	0,6	9,45
44	15,68	53,34	16,84	68,28	77,32	1,2	8,78
18,19	9,02	8,67	25,76	32,77	1196,96	0,39	3,2
18,19	16,49	8,67	96,94	82,78	208,51	0,18	3,92
31,11	18,1	29,78	130,73	6889,18	144,63	0,39	4,63
31,11	15,68	8,67	150,89	299,68	633,61	0,03	3,2
59,89	7,74	135,73	22,51	3016,33	1584,48	0,68	12,25
51,71	6,6	88,91	16,54	2168,5	477,6	0,37	4,24
42,47	25,21	8,67	123,53	778,3	318,59	0,5	10,46
25,75	10,3	8,67	8,3	32,77	8,59	0,06	0,09
22,4	11,56	8,67	74,83	32,77	1082,24	0,18	4,63
63,26	15,2	144,05	40,93	118,87	78,51	0,49	3,25
25,75	9,88	8,67	42,51	32,77	4413,31	0,03	12,12
42,47	11,56	8,67	32,84	350,57	1354,6	0,18	5,34
68,57	38,62	75,35	199,37	6935,84	1521,88	1,59	29,02
22,4	15,68	8,67	57,94	371,38	287,77	0,18	0,93
25,75	12,39	8,67	21,85	0,59	1346,97	0,03	5,69
50,75	27,54	8,67	227,3	13506,91	10128,99	0,91	40,98
39,17	13,63	29,78	55,95	52,06	10417,89	0,29	29,33
51,71	8,32	106,86	9,39	24,88	602,04	0,37	3,58
55,39	23,65	46,59	183,9	15604,34	5584,8	1,2	29,64
25,75	9,88	8,67	129,32	32,77	2999,53	0,18	8,78
42,54	6,18	101,23	11,3	8,99	86,83	0,27	0,79
45,46	35,21	8,67	258,57	53889,73	313,68	0,71	11,12
9,095	9,02	8,67	62,24	481,14	195,66	0,03	1,71
18,19	9,02	8,67	27,04	268,78	12,3	0,06	0,93
25,75	11,56	8,67	19,83	120,5	1602,93	0,39	7,42
55,39	49,85	46,59	585,52	6938,07	3903,45	1,59	24,96
62,62	13,06	126,85	126,47	94,39	3376,45	0,52	18,14
18,19	24,43	8,67	144,17	9575,15	1174,96	0,39	5,34
59,89	9,48	126,85	17,2	1157,47	329,08	0,42	2,61
66,32	14,13	139,95	151,59	159970,94	586,12	0,87	8,06
78,2	33,15	151,92	273,11	3582,07	8088,02	1,29	35,23
49,5	39	29,78	699,72	19340,32	11105,61	1,01	44,01
72,72	15,82	153,82	69,4	170,47	983,34	0,79	7,34
25,75	9,88	8,67	11,47	32,77	116,35	0,03	0,52
53,14	39	29,78	377,39	2167,89	1968,56	0,81	17,01

SCGF_b_CVL	SDF_1a_CVL	TNF_b_CVL	TRAIL_CVL	IL_1b_SC	IL_1ra_SC	IL_2_SC	IL_4_SC
19,805	39,15	0,75	4,9				
19,805	87,72	1,55	44,54				
19,805	81,62	1,15	40,54				
19,805	47,28	0,54	6,05				
19,805	39,15	0,75	16,96	515,08	14406,29	15,01	0,78
19,805	81,62	2,92	15,9				
19,805	68,8	0,54	9,41	140,58	45358,95	4,05	0,67
19,805	572,01	2,14	40,38	334,55	24863,25	4,63	1,25
19,805	417,06	1,44	5,08	530,77	1531,19	1,22	1,84
19,805	93,64	1,15	54,44	2486,98	22096,36	16,57	6,87
19,805	39,15	0,54	7,18				
19,805	54,85	0,54	11,6	595,57	24273,38	6,16	0,78
19,805	417,06	2,76	18,73	8,31	31,58	0,79	1,55
19,805	39,15	0,54	2,53				
19,805	115,9	1,95	20,12				
19,805	178,57	2,53	144,65	2665,84	33497,01	11,66	1,85
19,805	19,91	1,45	18,01				
19,805	224,92	0,54	3,73	1754,89	41324,26	16,87	1,51
19,805	397,53	1,55	120,98				
19,805	81,62	1,65	20,12				
19,805	235,99	2,34	2	2240,57	33384,17	13,25	2,72
779,34	425,68	1,55	223,48				
				393,88	18419,01	2,05	1,29
19,805	54,85	0,75	4,9				
19,805	340,92	1,94	0,17	1140,7	1594,32	18,18	3,68
19,805	115,9	1,75	133,31				
19,805	39,15	0,11	9,41	75,02	41647,82	4,23	0,91
19,805	54,85	0,54	9,41				
19,805	54,85	1,55	18,01				
19,805	178,57	5,57	452,76				
19,805	381,19	2,34	22,94	57,68	2393,43	0,79	2,02
19,805	105,02	1,15	95,21				
19,805	433,74	3,02	6,15	1,66	895,11	1	1,03
19,805	629,02	4,04	28,45	72,36	846,96	2,05	1,66
19,805	521,56	2,81	218,7	2832,91	2881,03	15,59	3,11
19,805	182,98	2,34	239,36				
19,805	433,74	3,07	34,68	48,85	33680,26	5,81	1,62
19,805	54,85	1,15	11,6	18,13	33250,8	3,86	0,57
19,805	136,46	0,95	124,77				

IL_5_SC	IL_6_SC	IL_7_SC	IL_8_SC	IL_9_SC	IL_10_SC	IL_12p70_SC	IL_13_SC	IL_15_SC
3,52	11,91	11,08	614,62	6,34	23,98	87,71	2,38	10,39
6,13	15,1	6,99	533,8	9,91	33,49	138,86	1,84	7,78
0,38	67,25	3,69	6797,79	9,91	19,01	129,62	6,49	1,41
0,45	5,29	1,92	2547,66	3,73	2,39	16,72	2,68	0,3
4,6	8,08	13,3	2100,52	11,37	49,17	130,18	6,37	0,0001
2,81	5,1	7,85	768,51	13,04	31,64	114,27	2,7	0,0001
0,1	0,58	1,06	21,65	1,94	3,32	31,64	2,98	0,76
7,96	77,17	13,44	966,48	12,96	37,63	175,04	4,33	0,0001
1,21	10,26	13,82	12123,32	25,3	33,15	192,2	3,06	14,27
0,74	51,7	7,71	57063,94	10,01	23,77	271,88	15,73	3,36
1,3	32,03	5,04	7947,07	3,77	5,59	41,89	7,63	1,02
0,51	568,26	3,1	963,92	7,3	16,65	22,83	4,49	2,64
5,2	44,37	14,85	815,87	10,05	31,39	115,15	3,06	0,0001
0,31	14,78	2,02	172,43	3,42	2,93	15,74	1,85	0,5
0,17	0,65	1,16	22,51	1,84	2,55	10,88	1,29	1,46
0,35	33,77	3,98	1156,8	2,44	2,23	14,77	1,85	1,51
0,63	711,61	4,51	12398,08	5,09	10,21	68,57	8,94	3,13
0,31	48,86	6,47	31084,45	6,78	9,55	120,39	8,39	5
0,56	4,21	3,1	283,25	7,73	27,54	89,95	1,27	18,74

IL_17_SC	Eotaxin_SC	FGFbasic_SC	G_CSF_SC	GM_CSF_SC	IFN_g_SC	IP_10_SC	MCP_1_SC
39,55	5,43	25,51	2741,51	107,85	40,14	32,83	22,22
31,82	0,205	19,42	3907,45	105,96	26,03	441,54	37,61
50,72	30,79	12,38	1225,4	17,2	67,62	5874,39	46,82
74,47	17,45	43,62	99,68	28,87	86,57	2908,2	30,57
85,22	25,49	52,44	787,43	134,2	297,58	2063,88	38,66
53,49	0,205	39,77	459,87	130	36,96	312,42	13,83
136,65	14,81	59,18	12,09	28,87	32,16	41,74	3,74
174,26	11,83	32,43	10767,05	118,24	188,63	1036,45	74,11
686,04	17,02	37,26	1839,06	93,48	84,66	169509,46	102,64
28,03	54,94	24,29	3678,4	39,4	176,66	16912,99	23,74
6,22	17,01	13,99	795,39	31,1	72,82	1530,32	99,9
176,12	37,53	68,84	13655,98	32,66	209,2	118,95	62,89
56,41	2,62	27,4	2649,34	119,87	44,84	440,78	129,56
114,35	21,8	57,34	222,87	26,65	53,62	45,63	4,79
82,98	17,45	36,71	20,5	36,48	37,6	1342,47	4,12
102,42	21,37	39,2	2128,11	28,2	65,88	107,86	142,67
51,49	47,11	41,2	7399,55	23,55	627,32	10423,22	49,61
62,13	37,53	19,32	1088,25	38,28	98,5	68702,05	129,67
34,41	0,205	24,06	280,36	143,04	22,63	27258,37	38,83

MIP_1a_SC	PDGF_bb_SC	MIP_1b_SC	RANTES_SC	TNF_a_SC	VEGF_SC	IL_1a_SC	IL_2Ra_SC
4,07	84,3	7,95	12,24	23,9	1009,76	840,89	78,25
1,89	28,53	7,35	7,7	17,35	3244,44	1089,09	60,49
1,67	33,89	384,8	22,31	63,34	3578,2	235,75	182,04
1,48	46,45	17,12	5,18	28,61	597,7	156,95	97,22
4,59	79,66	55,92	13,77	140,64	3625,13	6408,85	76,31
4,19	32,79	9,11	13,52	28,8	2151,27	276,72	41,72
1,45	5,88	4,66	4,34	7,15	916,08	193,51	38,54
4,98	61,22	37,34	51,79	86,61	6681,34	1373,28	150,54
4,11	132,52	55,92	90,42	35,59	8632,1	892,1	84
2,8	42,71	68,26	40,51	150,92	17203,15	838,71	133,64
0,89	32,37	10,56	17,29	25	706,95	266,26	75,63
5,91	28,66	365,93	7,29	1043,29	689,32	192,64	44,37
2,02	81,21	10,21	11,15	20,9	1255,56	1000,45	102,7
1,46	9,48	17,47	4,03	15,59	338,47	100,4	38,54
1,42	35,92	3,37	5,08	5,59	600,23	227,85	34,65
1,58	70,24	10,56	3,42	26,58	423,86	421,78	91,33
2,5	42,54	12,14	16,57	336,23	2458,47	1948,31	239,53
0,95	80,39	22,53	93,15	11,18	2885,86	152,94	95,25
0,94	47,35	5,07	14,25	8	1231,82	162,23	68,48

IL_3_SC	IL_12p40_SC	IL_16_SC	IL_18_SC	CTACK_SC	GROa_SC	HGF_SC	IFN_a2_SC	LIF_SC
111,47	210,85	49,79	1996,36	50,87	104,83	803,53	50,75	65,68
90,94	123,56	52,53	406,2	36,81	173,62	614,82	39,17	49,48
460,43	519,42	197,93	6301,63	62,36	592,92	3240,88	105,44	41,86
219,09	176,39	99,69	1984,98	35,38	730,64	381,28	79,05	26,35
148,1	194,83	80,85	2272,96	71,09	496,56	2065,95	55,39	68,59
70,68	2,825	37,76	61,79	48,29	65,27	1174,23	25,75	55,03
99,11	87,5	56,13	764,26	18,8	356,9	62,19	61,28	21,17
216,17	627,7	136	14947,76	89,71	170,28	3369,06	82,31	118,25
187,89	566,66	87,06	1322,95	62,65	1781,41	1013,83	59,56	30,63
451,44	397,81	149,73	3056,31	61,42	666,29	784,88	104,18	50,43
223,67	186,78	110,48	3726,56	33,68	398,65	668,66	81,49	42,89
101,78	67,37	70,2	276,19	18,8	407,01	1986,05	65,13	33,83
164,9	587,3	95,95	3328,36	71,09	645,95	675,97	65,16	42,01
99,11	80,77	47,13	236,32	14,52	314,35	353,86	58,43	8,03
81,43	95,95	56,13	15,27	11,43	362,11	47,26	58,43	8,03
186,49	166,03	85,99	3172,14	38,16	464,72	1469,4	80,69	43,59
402,94	358,73	189	1966,99	67,47	848,61	8597,06	100,24	105,69
269,64	235,48	116,32	1834,47	43,53	765,51	891,29	86,71	25,53
142,3	423,71	62,77	742,24	53,37	1495,7	68,82	49,5	20,49

MCP_3_SC	M_CSF_SC	MIF_SC	MIG_SC	b_NGF_SC	SCF_SC	SCGF_b_SC	SDF_1a_SC
38,93	839,72	120,5	635,92	1,01	11,46	19,81	131,45
59,46	1045,66	5255,88	4685,03	0,6	17,98	19,81	105,02
206,88	278,38	6013,94	85445,96	3,17	103,44	4393,46	817,11
166,52	187,97	5566,13	5808,14	1,41	38,77	19,81	583,89
29,78	1347,31	1055,17	4859,76	2,15	22,44	68,1	174,11
8,67	337,59	108,65	3936,54	0,03	11,46	19,81	87,72
124,53	297,72	886,22	64,34	0,57	2,61	19,81	508,09
122,1	1717,55	354,08	7015	3,97	51,82	19,81	310,96
97,06	1193,38	2062,39	41974,7	1,59	137,93	19,81	260
224,31	280,6	5307,89	85445,96	3,01	108,3	1356,08	756,68
159,39	187,49	2286,09	2823,73	1,27	22,65	39,61	559,86
95,27	392,32	1192,46	3818,81	0,68	15,07	19,81	508,09
80,07	806,95	8681,86	2450,81	1,59	27,46	19,81	182,98
101,23	96,88	158,96	2076,81	0,37	8,06	19,81	340,92
126,85	574,97	37,95	3887,6	0,37	16,99	19,81	340,92
131,36	997,78	4578,02	4174,02	1,74	22,06	4785,98	629,02
213,76	776,75	977,48	85445,96	2,63	119,26	2377,08	833,59
166,52	431,24	2028,95	10501,06	1,59	55,39	19,81	606,9
88,89	585,66	1601,91	19683,45	1,01	73,38	19,81	165,05

TNF_b_SC	TRAIL_SC
3,68	60,34
3,3	72,05
4,81	235,75
3,5	178,42
6,32	281,31
2,43	46,53
2,24	27,22
5,94	435,03
4,25	116,22
4,81	465,58
2,19	148,89
1,25	48,73
2,72	125,72
1,84	12,29
2,14	15,19
3,5	67,14
6,4	321,75
3,28	77,87
4,06	56,41
